# 14.1 T Liquid-State ^19^F Overhauser Dynamic Nuclear Polarization in an Analytical Organic Setting

**DOI:** 10.1021/jacs.6c03789

**Published:** 2026-05-07

**Authors:** Sungsool Wi, Jenica Lumata, Thierry Dubroca, Tomas Orlando, Lucio Frydman

**Affiliations:** † 189689 National High Magnetic Field Laboratory, Tallahassee, Florida 32310, United States; ‡ Department of Chemical and Biological Physics, 34976 Weizmann Institute of Science, 7610001 Rehovot, Israel

## Abstract

Liquid-state ^19^F Overhauser dynamic nuclear polarization (DNP) was applied to several organic compounds using 1,3-bis­(diphenylene)-2-phenylallyl (BDPA) as the polarizing agent. ^19^F DNP enhancement factors ranging between 3- and 37-fold were achieved upon microwave irradiation at 395 GHz/14.1 T. These results were obtained using a conventional liquid-state NMR probehead, modified to incorporate a waveguide for the microwaves capable of delivering ≈13–30 W of power emanating from a gyrotron source, as well as a field-sweeping coil for precise tuning of the Overhauser effect resonance condition. Experiments were conducted on ≈70 μL sample volumes, yielding spectral resolutions that were comparable to those of standard liquid-state ^19^F NMR. These studies were complemented with quantitative determinations of BDPA’s T_1e_ and T_2e_ relaxation times in these liquids, using pulsed-echo and inversion–recovery measurements at 94 GHz. From these data, saturation factors, leakage factors and BDPA coupling factors, could be extracted for each fluorinated system. The resulting coupling factors ranged from −0.0067 to −0.14, depending on the analyte and solvent system used. While heating issues remain to be resolved, these results open new vistas for performing high-field ^19^F DNP NMR on organic liquids, on sample volumes of relevance in chemical analysis. Such approaches could significantly broaden the applicability of liquid-state ^19^F DNP for small-molecule NMR in analytical and pharmaceutical chemistry, and eventually for the detection and characterization of environmentally persistent PFAS compounds.

## Graphical abstract

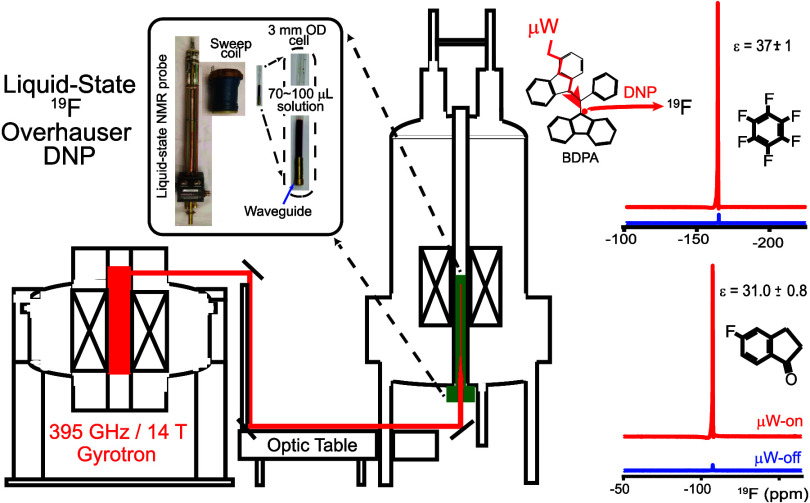

## 1 Introduction

NMR has long been a central analytical tool for investigating the structure and dynamics of molecules across diverse scientific disciplines, including chemistry, materials science, and biology.^1–4^ Despite an exceptional versatility and broad applicability, NMR’s use is often limited by an inherently low sensitivity resulting from the small population differences between the spin states involved. This limitation often necessitates relatively large sample amounts or extended signal averaging times. The use of specialized methods to enhance sensitivity, such as dynamic nuclear polarization (DNP),^5–9^ spin exchange optical pumping,^10,11^ para-hydrogen induced enhancements,^12–14^ or photochemically-induced DNP,^15–17^ is also gaining increasing acceptance for sensitizing these experiments. Most general among these NMR sensitivity-boosting strategies is DNP, as it enhances NMR signal intensity by transferring to nearby nuclear spins the much higher polarization of unpaired electron spins. DNP transfers are typically mediated through scalar or dipolar electron–nuclear interactions involving stable radicals or paramagnetic centers, leading to maximal theoretical enhancements on the order of |*γ*_*e*_/*γ*_*n*_|. Here *γ*_*e*_ and *γ*_*n*_ are the gyromagnetic ratios of the electron and the nucleus of interest, respectively, meaning that DNP can yield sensitivity enhancements of up to ∼700-fold for ^1^H and ^19^F, and of several thousand-folds for ^13^C and ^15^N.^18^ Since NMR’s sensitivity improves with the square root of the number of averaged scans, these enhancements correspond to a potential reduction in signal averaging time by factors of millions.

Cryogenic DNP—particularly under solid-state magic-angle-spinning or in combination with sudden sample melts and dissolutions—has become increasingly widespread across a broad range of structural, materials, and biomedical NMR applications.^8,19–32^ These solid-state and hyperpolarized dissolution approaches have delivered dramatic sensitivity gains and enabled high-impact studies; however, they are inherently constrained by their cryogenic operation to frozen pellets and, for dissolution DNP, to single-shot measurements subject to substantial sample dilution. These limitations have motivated continued efforts toward developing robust, multiscan DNP strategies directly compatible with conventional liquid-state NMR. Liquid-state DNP NMR has been extensively demonstrated at low magnetic fields via the Overhauser mechanism,^18,24,28,33–40^ but its extension to high magnetic fields remains challenging.^41–48^ In particular, dipolar-based ^1^H Overhauser DNP (ODNP) becomes increasingly inefficient with field strength, and while specialized microcavity probes and particle-relayed schemes have demonstrated feasibility,^49–58^ these approaches remain limited in generality, sample volume, and/or practical implementation. As a result, robust, high-field, multiscan ^1^H DNP in the liquid state is still not available as a widespread analytical technique.

These limitations have motivated the exploration of alternative nuclear targets for liquid-state DNP at high field, among which ^19^F is a particularly attractive candidate. Although ^19^F NMR is arguably less universally applicable than ^1^H counterparts, it offers several advantages that could make it into a powerful analytical tool. The ^19^F nucleus has a 100% natural abundance, partakes of numerous pharmaceutically and environmentally important chemical structures, and exhibits a wide chemical shift dispersion, which enables high spectral resolution and facilitates detailed structural and dynamic analyses.^59^ These attributes position DNP-enhanced ^19^F NMR as an attractive analytical approach. Numerous reports have successfully demonstrated DNP-enhanced ^19^F NMR at relatively low (0.35–3.4 T) and high (5–9.4 T) magnetic fields;^47,48,60–69^ studies have also shown high-field ^19^F DNP in the solid state.^70–74^ The technique promises a considerable applicability in high-field liquid-state investigations, thanks to electron–^19^F scalar couplings capable of supporting efficient ^19^F DNP even at high Larmor frequencies.^36,68,69^ This is unlike the situation arising in ^1^H DNP, which relies almost exclusively on dipolar-coupling-based mechanisms that become ineffective in liquids at high magnetic fields.

Liquid-state ^19^F DNP investigations have identified TEMPO, galvinoxyl and their derivatives as effective polarizing radicals, particularly for aromatic ^19^F sites.^47,69^ This study expands these efforts, by exploring liquid-state ^19^F DNP using relatively large sample volumes (∼70 μL). To this end various organic small molecules were dissolved in nonpolar, low microwave-absorbing solvents, such as *p*-xylene-d_10_, toluene-d_8_ and CCl_4_. Measurements were then carried out at 14.1 T, the highest DNP NMR field assayed in liquids so far, and relied on DNP based on electron–^19^F scalar couplings, with BDPA serving as the polarizing agent. Compared to nitroxide-based radicals, BDPA offers several advantages, including a single, relatively narrow EPR line that avoids the splittings caused by nitrogen hyperfine couplings.^75–77^ This facilitates a more efficient, uniform microwave saturation. Additionally, BDPA exhibits relatively long high-field electron spin relaxation times T_1e_ and T_2e_, facilitating the microwave-driven electron saturation and an enhanced nuclear polarization build-up.^78–80^ BDPA is also inexpensive, relatively stable and hydrophobic, making it well-suited for organic solvents without suffering from rapid degradation or side reactions. Finally, being devoid of strong hydrogen bonding or proton exchanges, it minimizes extrinsic relaxation pathways and maximizes DNP’s cross-relaxation to nearby nuclei. When tested on ^19^F-containing samples at 14.1 T, significant signal enhancements of up to 37-fold were observed for a range of commercially available compounds and pharmaceuticals, particularly for aromatic fluorocarbons, which exhibited greater enhancements than their aliphatic counterparts. The implications of these results, the mechanisms underlying these large-sample/high-field enhancements, and potential extensions of this method for practical and scalable applications, are briefly discussed.

## 2 Experimental Section

All chemicals were obtained from Millipore-Sigma and used without further purification. The compounds studied include hexafluorobenzene (HFB); 2,4,5-trifluorobenzaldehyde (2,4,5-TFBA); fluorobenzene (FB); 4,4′-difluorobenzophenone (4,4′-DFBP); 3′,4′,5′-trifluoropropiophenone (3′,4′,5′-TFPP); 5-fluoro-1-indanone (5-FIA); 1H,1H,2H-perfluoro-1-hexene (PF-1-hexene); perfluorodecalin (PFD); 2-(trifluoromethyl)­quinoline (2-TFMQ); diethyl fluoromalonate (DEFMal); and dexamethasone (DEX). These ^19^F-containing molecules were selected to systematically investigate how molecular structure influences high-field liquids ^19^F DNP; particularly the differences between aliphatic and aromatic frameworks, the positional accessibility of radicals to the ^19^F sites (peripheral versus internally embedded), and structural features governing whether radical–analyte encounters are sterically favored or hindered. Tested solutions were prepared by dissolving 20–40 mM BDPA radical and 270–540 mM of the ^19^F-containing target molecule, in ∼70 μL of CCl_4_ or *p*-xylene-d_10_. Although for the latter solvent the ODNP enhancements were highest and kept on increasing with BDPA concentration, solubility problems prevented us from dissolving this radical at >40 mM. Each solution was transferred into a fluorinated ethylene propylene (FEP) tube (3 mm outer diameter, 2 mm inner diameter) and sealed by thermal welding. Prior to welding, all samples underwent at least six freeze–pump–thaw cycles under a nitrogen atmosphere to remove dissolved oxygen. The sealed tubes were then stored in a nitrogen-purged glovebox for several days to enable further diffusion and removal of residual oxygen through the plastic FEP wall.^81^ Although BDPA may degrade in solutions,^82^ under these conditions and over the duration of our experiments, we did not observe any effect that would hamper the measured enhancements. The FEP tubes did not show deformation or loss of shape despite their repeated use for ODNP NMR on different samples.

Liquid-state ^19^F DNP NMR experiments were performed using a 14.1 T Oxford NMR magnet coupled to a Tecmag Redstone NMR console configured for ^19^F detection at 565.1 MHz. Microwave (μw) irradiation at the second harmonic mode (395 GHz) was supplied by a Bruker-CPI gyrotron, with μw power and polarization guided and controlled via a quasi-optical bench.^38^ A fast-acting shutter synchronized with the pulse program sequence was used to rapidly open or close the μw beam path to the sample. While the gyrotron output was typically 30 W, the average μw powers used in the ODNP experiments presented here were approximately 13 W, as regulated by the quasi-optical setup.

The NMR probe employed was a modified Varian HX 600 MHz broadband 5 mm liquid-state probe, adapted with a customized sweep coil for targeting various radicals, as described in previous publications.^38,42,46,83^ The ^1^H channel of this probe was tuned to cover the ^19^F Larmor frequency used in the experiments. The 90° RF pulse length for ^19^F was 50 μs and the ensuing receiver deadtime ca. 200 μs; this delay is longer than FEP’s T_2_ decay time (≈20 μs), and hence no tube background was detected. Chemical shift scales were referenced using the C_6_F_6_ peak, resonating δ = −164 ppm relative to CFCl_3_ = 0 ppm. A saturation-recovery pulse followed by a Hahn echo sequence (inserted because of the relatively long deadtimes and used also in the regular DNP experiments) was used for the T_1_ measurements. The saturation recovery delay time was also employed as the μw-on period (shutter open), while the μw beam was blocked (shutter closed) during all other intervals –including the acquisition delay d_1_. The μw-on :μw-off time ratio was maintained at 1:6∼1:8 throughout all DNP experiments, to allow sufficient cooling time after microwave sample heating. All experiments were performed after shimming the magnetic field using a 10 v/v% HFB solution in *p*-xylene-d_10_. DNP buildup curves were acquired with 2–4 scans, and the resulting data were directly used for buildup analysis. In contrast, ^19^F T_1_ measurements performed in the absence of microwave irradiation—on both pure samples without BDPA and DNP samples containing BDPA—were acquired using 16–32 scans to improve sensitivity. Analyte concentrations were kept identical for samples with and without BDPA. Saturation-recovery experiments employed 80 consecutive 90° pulses with a 1 ms delay for spin saturation.

Both the radical concentration and the analyte solute concentration were optimized for ^19^F Overhauser DNP using HFB in *p*-xylene-d_10_ as test sample, and the results are provided in the Supporting Information (Figure S1). Based on these measurements, the optimal conditions were identified to be approximately 40 mM BDPA and 540 mM HFB, conditions that are like those reported by Reinhard et al.^48^ All subsequent DNP experiments on the other compounds were conducted using these concentrations as guiding reference values.

A continuous flow of N_2_ gas at 30 L min^–1^ was supplied by an FTS temperature control unit (SP Industries Inc.), and delivered through the microwave waveguide from the bottom of the probe into the sample compartment to ensure efficient temperature regulation. This continuous N_2_ flow also maintained an oxygen-free environment around the sample cell during the DNP experiments. To determine the extent of sample heating caused by microwave irradiation during the DNP experiments, a temperature-calibration study was performed under an off-Overhauser Effect (off-OE) condition (ω_0_(^19^F) = 564.965 MHz; ω_0_(^1^H) = 601 MHz; ω_0_(^13^C) = 151.2 MHz). The temperature calibration sample consisted of approximately 10 v/v% ^13^CCl_4_ and ^13^CHCl_3_ dissolved in *p*-xylene-d_10_, together with 40 mM BDPA. BDPA was included in order to reproduce the actual DNP experimental conditions as closely as possible. Temperature changes in the sample were monitored by tracking the variation in the ^13^C chemical-shift difference between ^13^CCl_4_ and ^13^CHCl_3_ as a function of microwave-irradiation time.^42^ For example, with 40 s of microwave irradiation (followed by a cooling period eight times longer at an FTS setting of −30 °C with a N_2_ flow rate of 30 L min^–1^), the sample temperature rose to approximately 150 °C. It has been reported that, under pressurized conditions, a superheated state above the solvent’s boiling temperature can still be maintained in the liquid phase;^54^ we observed no evidence of sample boiling. Additional details of the temperature-calibration experiments are provided in the Supporting Information (Figure S3).

Ancillary 395 GHz continuous-wave (CW)­EPR spectra were recorded using a home-built transmission-type spectrometer without a resonant cavity.^84^ The superconducting magnet used for this (Oxford Instruments) can generate magnetic fields up to 16 T. The microwave source consisted of a phase-locked oscillator (Virginia Diodes) operating at a tunable fundamental frequency of 8–20 GHz, followed by a frequency-multiplier chain to generate higher harmonics. A slow magnetic-field sweep rate of 0.02 mT s^–1^ was employed, with the field-modulation amplitude carefully minimized until no observable distortion of the spectral lineshapes was detected. CW EPR spectra were fitted with the Matlab package Easyspin and the routine *garlic*.^85^

To measure its T_1e_ and T_2e_, BDPA was dissolved in 30 μL of degassed toluene-d_8_ at a 40 mM concentration and loaded into FEP tubes. The electron spin relaxation times were then measured at 94 GHz (3.35 T) using the high-power, high-field pulsed EPR spectrometer (HiPER) located at the National High Magnetic Field Laboratory (NHMFL, Tallahassee, Florida, USA). This spectrometer is a duplicate of the instrument developed by the group of G. Smith at the University of St. Andrews.^86^ T_2__e_ was determined using a variable-delay Hahn echo pulse sequence, while T_1__e_ was measured by preceding a Hahn echo sequence with an initial saturation pulse followed by a variable recovery delay. Owing to the high peak microwave power available on the HiPER system (>1000 W), 90° pulses of 25 ns duration were employed. The sample temperature was maintained at 27 °C using a helium gas flow. Although high peak powers were used, the average microwave power delivered to the sample was low (≈0.5 W), because of the long recycle delays (>100 μs) imposed by the high-power microwave amplifier. This low average power, in combination with active temperature control, ensured a stable sample temperature throughout the experiments. While these frequencies and temperatures were not identical to the ones used to carry out the DNP NMR experiments, they could still provide valuable data to extrapolate the degree of saturation of BDPA’s electron spin transition at high fields.^89^

The ODNP signal buildup curves were fitted using the functional form^1,2^

$$S\left(t\right)=A\left(1-me^{-tR_{B}}\right)$$ 1

where *A* is the maximum enhancement amplitude corresponding to the plateau region of the buildup curve, *m* ≤ 1 is an empirical scaling factor introduced to improve the quality of the fit, and *R*_*B*_ is the DNP signal buildup rate (see Supporting Information for details). Although this expression deviates from the idealized form *A*(1 – *e* ^–*tR*_*B*_^), it remains a single-exponential function, and the extracted *R*_*B*_ values retain their proper physical meaning. Such a modified fitting form can arise when the saturation recovery pulse block does not destroy completely the initial magnetization, leaving a residual nonzero longitudinal component.^1,2^ In our experiments, saturation was achieved by repeating a block consisting of a 90° pulse followed by a 1 ms delay, 80∼120 times. The recovery experiment—during which the microwave irradiation was also applied—was then carried out with variable recovery times. Even at zero recovery time, a very small residual signal remained, causing the buildup curve to start from a nonzero value. To account for this common DC offset, a constant baseline elevation was uniformly subtracted from all data points prior to fitting, after which the buildup curves were analyzed using eq 1.

## 3 Results

### 3.1 Overall ^19^F DNP features

Figure 1A shows the ^19^F ODNP field-sweep profile obtained for a 70 μL solution containing 40 mM BDPA and 540 mM hexafluorobenzene (HFB) in *p*-xylene-d_10_. The sweep was performed by varying the current through a home-built sweep coil (−0.15 A to +0.15 A), corresponding to a frequency range of 564.95–565.20 MHz (14.10541–14.11165 T) around the ^19^F Larmor frequency 565.083 MHz in our nominal “14.1 T” NMR magnet. The resulting DNP frequency sweep profile maximized at a ^19^F frequency of 565.083 MHz, with a peak width at half height (PWHH) of approximately 1.497 mT (60 kHz in ^19^F units) and a baseline width of about 2.5 mT (100 kHz for ^19^F)—matching roughly the EPR line width measured for BDPA at 395 GHz (see Figure 2). Figure 1B presents the μw-on and μw-off signal buildups measured at the optimal DNP field (565.083 MHz for ^19^F; 14.10874 T), for two different μw field strengths (B_1e_ = 0.063 mT and 0.04 mT).^42^ For these experiments, a ^19^F Hahn-echo detection sequence (τ = 500 μs) was preceded by a saturation-recovery block consisting of 80 90° ^19^F RF pulses separated by a 1 ms delay, and a variable recovery period. This variable recovery period also served as the microwave-irradiation (μw-on) time. To prevent sample overheating, a recycling delay eight times longer than the saturation-recovery (μw on) time, was applied between successive scans. The longitudinal relaxation rate in the absence of microwave irradiation was *R*_1_ = 0.91 ± 0.08s^-^^1^, whereas the polarization buildup rates under microwave irradiation were *R*_*B*_ = 0.27 ± 0.02s^^-^1^ and 0.19 ± 0.03s^^-^1^ for B_1e_ of 0.063 mT and 0.40 mT, respectively. Despite the slow, R_B_ < R_1_ polarization buildup rates under microwave irradiation, both *B*_1*e*_ field strengths yielded comparable DNP enhancements of *ε* = *I*_on_/*I*_off_ = 37 ± 1; this suggests that the saturation of the BDPA radicals was not a limiting factor under these conditions. Under on-OE conditions, microwave irradiation increased the ^19^F PWHH from approximately 40 Hz to about 120 Hz; under off-OE conditions, PWHH were ≈120–130 Hz due to limitations in our field-sweep coil setup, and only broadened by another ∼10 Hz upon microwave irradiation. In all cases, resonances remained relatively sharp even under all conditions (Figure 1C).

**Figure 1:**
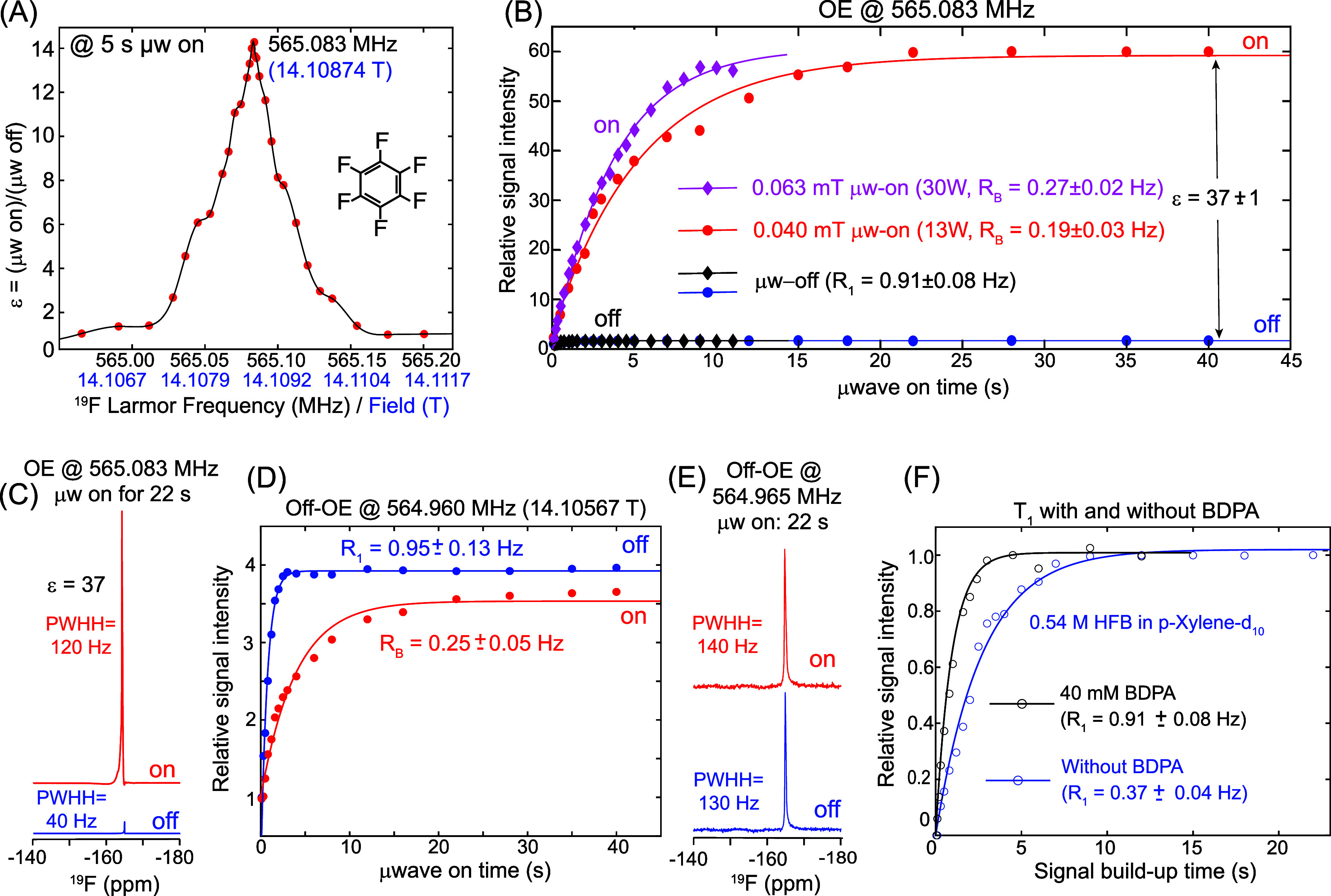
^19^F liquid-state Overhauser DNP (ODNP) results obtained for 540 mM hexafluorobenzene (HFB) and 40 mM BDPA codissolved in 70 μL of *p*-xylene-d_10_. (A) Field profile of the ^19^F Overhauser DNP enhancement, recorded by varying the magnetic field while irradiating a constant 395 GHz microwave beam for 5 s. The maximum enhancement occurs at a ^19^F Larmor frequency of 565.083 MHz, with the center of the DNP resonance profile showing a peak width of ∼60 kHz at half height (PWWH) and of ∼100 kHz at the baseline. (B) DNP build-up curves obtained by varying the microwave irradiation time at the optimal OE frequency (565.083 MHz) using 0.04 mT (red) and 0.063 mT (pink) microwave powers. The corresponding microwave-off (black and blue) data are also shown. A maximum signal enhancement of ε = 37 ± 1 was achieved. The 0.63 G curve was truncated at 11 s due to sample heating. (C) Representative ^19^F spectra obtained under optimal OE conditions (565.083 MHz) with and without microwave irradiation (22 s, on/off). (D) Microwave on/off build-up curves recorded at an off-OE frequency (564.965 MHz), where no enhancement is observed, confirming that the signal increase in (B) arises solely from the Overhauser effect. (E) Corresponding on/off ^19^F spectra at 564.965 MHz showing no enhancement. (F) ^19^F spin–lattice T_1_ relaxation build-up curves measured in the absence of microwave irradiation, comparing solutions with and without 40 mM BDPA. The addition of BDPA shortens T_1_ from 2.7 s (R_1_ = 0.37 Hz) to 1.1 s (R_1_ = 0.91 Hz), consistent with enhanced relaxation pathways introduced by the radical.

**Figure 2:**
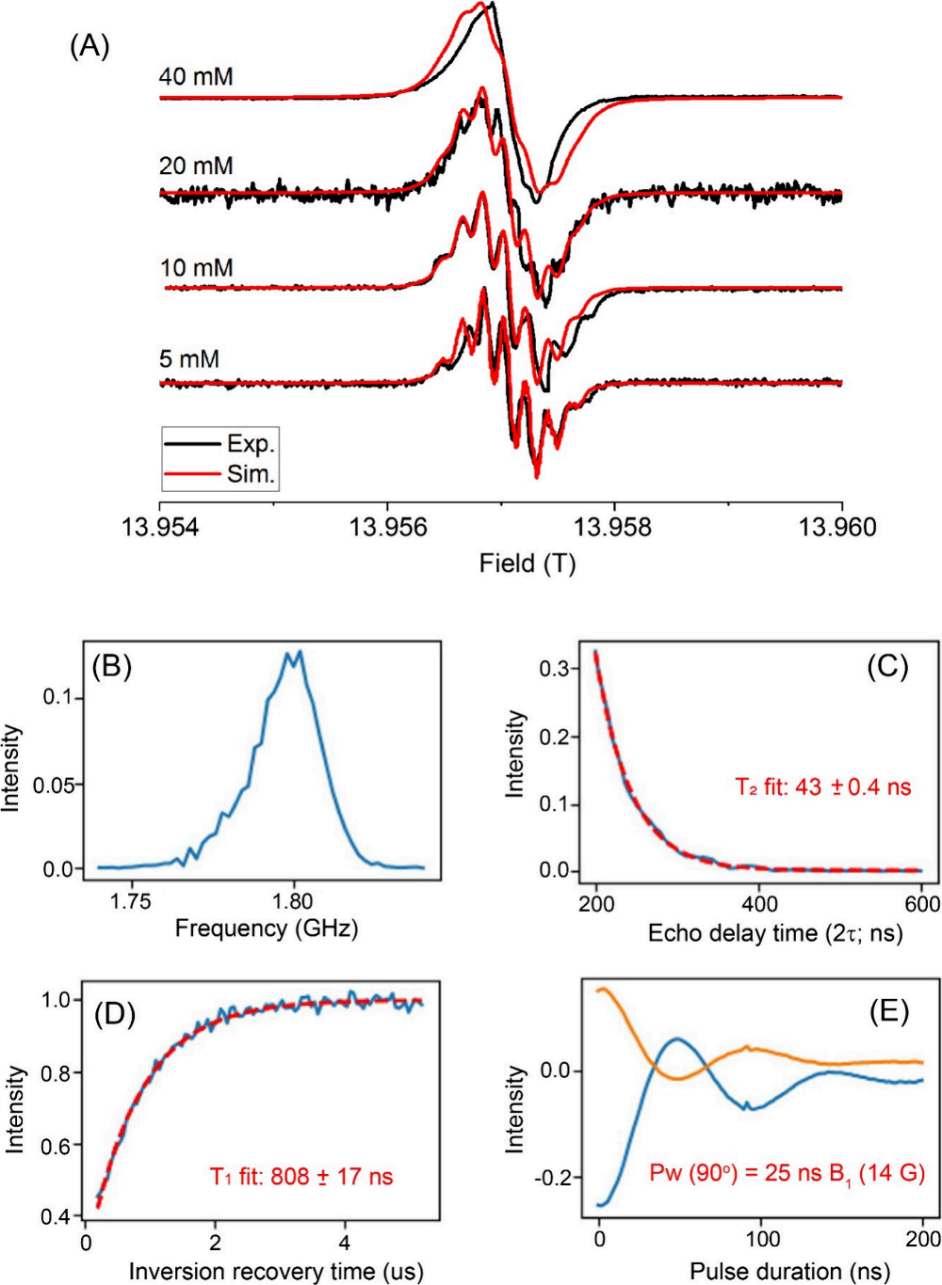
EPR spectral and relaxation measurements of BDPA radical solutions in *p*-xylene-d_10_. (A) Experimental and simulated (EasySpin) CW EPR spectra of BDPA at 5, 10, 20, and 40 mM concentrations. The electron–^1^H hyperfine structure is visible up to 20 mM, while line broadening dominates above 40 mM. (B–E) Pulsed EPR measurements performed on a 30 μL sample of 40 mM BDPA in toluene-d_8_ at 300 K using the 94 GHz HiPER spectrometer operating in Hahn-echo mode. (B) Frequency-swept EPR profile recorded by monitoring the integrated echo intensity across the resonance field. (C) Spin–spin relaxation decay measured by varying the echo delay time (2τ), yielding T_2_e = 43.0 ± 0.4 ns. (D) Spin–lattice relaxation (T_1e_) determined from an inversion–recovery experiment, giving T_1e_ = 808 ± 17 ns. For all pulsed experiments, the π/2 and π pulse lengths were approximately 20 and 40 ns. (E) Real and imaginary components of the microwave nutation curves showing decay due to relaxation. From these nutation curves, the π/2 and π pulse lengths were determined to be approximately 20 and 40 ns, respectively.

As shown in Figure 1D, an off-Overhauser Effect (off-OE) condition located outside the region of DNP enhancement (Figure 1A) exhibits no significant signal change – in fact, the μw-on signal is slightly smaller than the μw-off signal due to thermal effects. The saturation recovery buildup rate under μw-on, off-OE irradiation is significantly slower than the intrinsic longitudinal relaxation rate measured without microwaves: R_1_ = 0.95 ± 0.13s^-^^1^, and R_B_^μw‑on^ = 0.25 ± 0.05 s^–1^. This could reflect a heat-induced *T*_1_ elongation driven by the microwave irradiation; specifically, to a decrease in the viscosity of the solvent that shortens molecular tumbling correlation times, placing the ^19^F deeper into the extreme-narrowing regime. Also noticeable upon comparing on-OE (Figure 1B–1C) and off-OE (Figures 1D–1E) measurements, is a small shift (≈10 Hz) in the ^19^F peak arising under on-OE microwave irradiation; we ascribe such shift mainly to a microwave-power-dependent shift arising from partial saturation of the electron transition (saturation factor <1).^87^ Notice that NMR lineshapes under the off-OE condition exhibit broader line widths (≈130–140 Hz) than their on-OE counterparts (≈40–120 Hz). This broadening under off-OE conditions does not originate from the microwave irradiation. Rather, it arises from the fact that establishing the off-OE condition required passing a current of approximately 280 mA through our sweep coil, (whereas the on-OE condition was achieved with ≈ 0 mA); this coil current degraded the magnetic field shim homogeneity, which in turn led to the observed line width broadening.

### 3.2 Saturation and Leakage Characteristics

Figure 1F presents ^19^F T_1_ relaxation results obtained without microwave irradiation for 540 mM HFB samples with and without 40 mM BDPA, allowing evaluation of the leakage factor and quantification of the contribution of electron–^19^F cross relaxation to the overall relaxation process. The measured relaxation rates were R_1_ = 0.37 ± 0.04 s^–1^ without BDPA and R_1_ = 0.91 ± 0.08 s^–1^ with BDPA, corresponding to a leakage factor of *f* = 1-R_1_(without BDPA)/R_1_(with BDPA) = 0.59, indicating that approximately 59% of the total ^19^F relaxation arises from interactions with unpaired electrons in the BDPA radicals.

Figure 2A presents experimental EPR spectra acquired at 395 GHz—the same microwave frequency used for DNP irradiation—and the corresponding EasySpin simulations, for samples maintaining a constant 0.54 M HFB concentration, but containing variable BDPA concentrations of 5, 10, 20, and 40 mM in *p*-xylene-d_10_. Detailed descriptions of the EasySpin simulations and the g-tensor parameters derived from the best-fit simulations are provided in Table S3 (Supporting Information, Section 7). Distinct electron–^1^H hyperfine splittings are visible at BDPA concentrations ≤ 20 mM, whereas the spectral features appear broadened and unresolved at 40 mM.^88^ The 40 mM BDPA sample, in which the hyperfine structure is washed out, exhibits the highest DNP enhancement (Figure S1). Figure 2B–2E display the T_1e_ and T_2e_ relaxation measurements obtained for a 40 mM BDPA solution prepared in toluene-d_8_, using NHMFL’s HiPER pulsed EPR spectrometer operating at 94 GHz. In this case, instead of estimating electron relaxation parameters from the EPR line width to obtain T_2e_ and subsequently deriving the product T_1e_T_2e_ from a microwave saturation curve, both T_1e_ and T_2e_ were independently determined using pulsed inversion recovery and spin–echo EPR techniques, respectively. The T_1e_ and T_2e_ values measured were 808 ± 17 ns and 43.0 ± 0.4 ns, respectively. Notably, these values are approximately twice as long as those measured using similar pulsed methods at 3.4T for ^15^N-Tempone-d_16_.^39^ The relaxation parameters obtained at 3.4 T can be used to calculate the electron saturation at 14.1 T under the assumption that T_1e_ shows a flat field dependence above ∼3 T,^89^ and that T_2e_ may decrease by a factor of ∼ 2 for radical concentrations of tens of mM.^82^ The saturation factor *s* can then be calculated as

2 $$s=\frac{\gamma_{e}^{2}B_{1e}^{2}T_{1e}T_{2e}}{1+\gamma_{e}^{2}B_{1e}^{2}T_{1e}T_{2e}}$$

For microwave *B*_1*e*_ field strengths of 0.04 mT and 0.063 mT (Figure 1B), these would then be approximately 0.63 and 0.81, respectively.

### 3.3 BDPA-Driven 14.1T ^19^F DNP NMR for Aromatic and Aliphatic Sites

Figure 3 surveys liquid-state ODNP results for a variety of molecules bearing aromatic ^19^F nuclei. All of these exhibit substantial signal enhancements under suitable microwave irradiation, consistent with the behavior observed for HFB. The enhancement factors (ε) are 28 ± 2 and 31 ± 2 for 2,4,5-TFBA (Figure 3B–C), 14.8 ± 0.3 for FB (Figure 3D), 29 ± 2 for 4,4′-DFBP (Figure 3E), 21 ± 1 and 14.8 ± 0.9 for 3′,4′,5′-TFPP (Figure 3F–G), and 31.0 ± 0.8 for 5-FIA (Figure 3H). Also in parallel with the case of HFB, all tested aromatic ^19^F sites—whether in mono-, di-, tri-, or polyfluorinated systems—showed an ODNP enhancement build-up kinetics that was slower than their spontaneous relaxation rates in the absence of microwaves; i.e., R_B_ < R_1_. The corresponding build-up/relaxation rate ratios R_B_/R_1_ varied between 0.1 and 0.2, indicating that polarization transfer from electrons to ^19^F nuclei proceeds more slowly than – and therefore it is probably capped by– the intrinsic nuclear relaxation. The large ODNP enhancements observed for aromatic ^19^F nuclei can be attributed to π–π stacking interactions between the aromatic rings and BDPA radicals.^48^ These interactions may reduce the electron–^19^F distance and/or increase their contact times, fostering partial orbital overlap that promotes scalar (Fermi-contact) coupling. The formation of relatively stable π–π adducts may also aid to enhancing lower-frequency spectral densities, and these adducts tumble more slowly than the isolated molecules (see Discussion).

**Figure 3:**
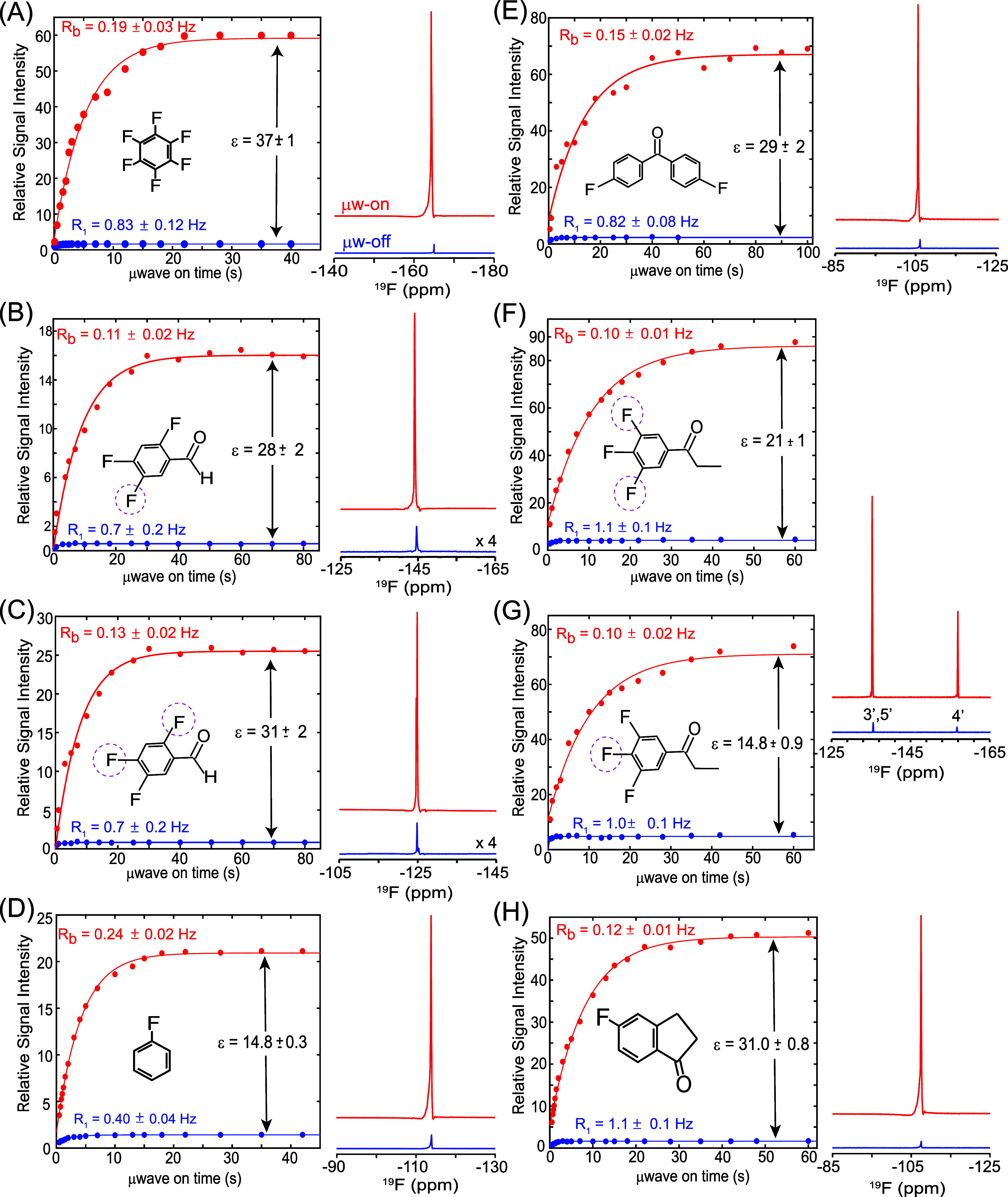
14.1T ODNP results obtained for ^19^F nuclei in aromatic systems. Shown are the liquid-state ^19^F ODNP buildup curves (left panels) and corresponding ^19^F NMR spectra recorded with μw-on (red) and μw-off (blue) (right panels) for: (A) HFB, (B–C) 2,4,5-TFBA, (D) FB, (E) 4,4′-DFBP, (F–G) 3′,4′,5′-TFPP, and (H) 5-FIA. For each sample, the buildup of DNP-enhanced ^19^F signal intensity was measured as a function of microwave irradiation time (t_μw_). These irradiation times were synchronized with saturation-recovery schemes; an 8-fold longer recycle delay (d_1_) was employed for each t_μw_ to maintain thermal stability and ensure sample cooling. The buildup curves (red circles) were fit to a single-exponential function with rate constants R_B_ and R_1_ representing the buildup and relaxation rates under μw-on and μw-off conditions, respectively. Significant ^19^F DNP enhancements (ε) were observed across the aromatic compounds, ranging from ε ≈ 15 ± 1 to 37 ± 1, as labeled in each panel.

Figure 4 presents liquid-state ^19^F ODNP results for several compounds containing fluorine atoms in aliphatic environments. These aliphatic ^19^F sites exhibit substantially smaller DNP enhancement factors ε than their aromatic counterparts. The measured DNP enhancement factors (ε) ranged from 3.4 to 14.2, depending on the molecular site and compound. These lower average enhancements might reflect the fact that ^19^F nuclei located in aliphatic functional groups do not engage in π-stacking interactions with BDPA, and therefore experience reduced electronic overlap and shorter electron–nucleus contact times (see Supporting Information for a DFT-based comparison of aromatic and aliphatic ^19^F sites). On the other hand, the build-up rates *R*_*B*_ associated with the aliphatic fluorine enhancements seem to be faster than their aromatic counterparts: whereas for aromatic ^19^F sites *R*_*B*_ ≈ (0.1∼0.3) ± (0.01∼0.03) s^–1^, similar analyses for the aliphatic ^19^Fs reveal *R*_*B*_ ≈ (0.38∼0.89) ± (0.01∼0.15) s^–1^. The R_B_/R_1_ ratios for the aliphatic sites were relatively close to one, ranging from 0.42 and 0.99. This might reflect the enhanced flexibility of aliphatic ^19^F moieties: these rates seemed to be less sensitive to temperature than their aromatic counterparts, leading to only minor differences under shorter and longer μw-on conditions and marginal T_1_ increases upon μw-induced temperature increases.

**Figure 4:**
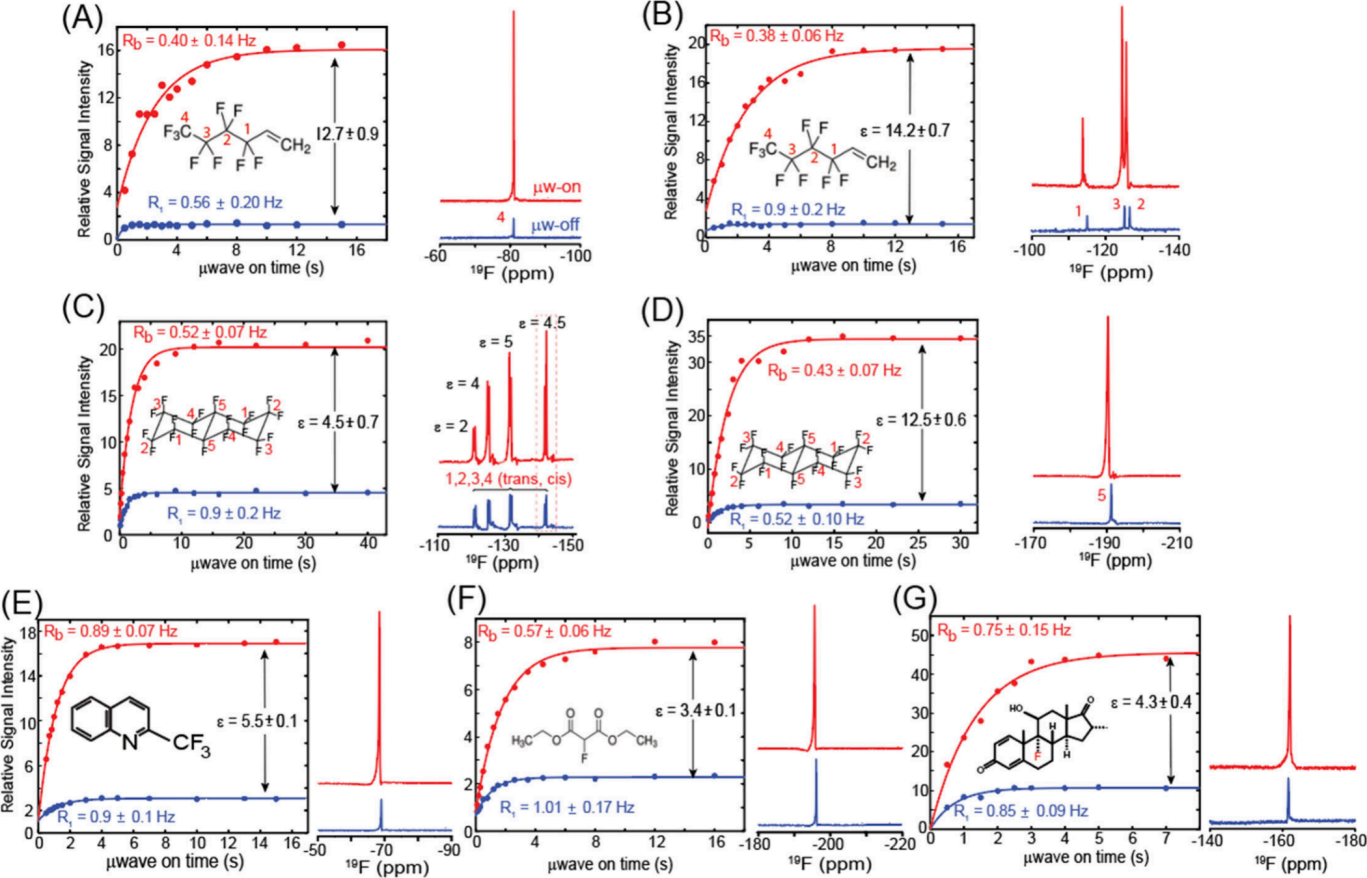
14.1T ODNP results obtained for ^19^F nuclei in aliphatic compounds. Shown are the liquid-state ^19^F ODNP buildup curves (left panels) and corresponding ^19^F NMR spectra recorded with microwave irradiation on (red) and off (blue) (right panels) for: (A, B) ^1^H,^1^H,^2^H-perfluoro-1-hexene, (C, D) perfluorodecalin, (E) 2-(trifluoromethyl)­quinoline, (F) diethyl fluoromalonate, and (G) dexamethasone. For each compound, the buildup of the DNP-enhanced ^19^F signal intensity was measured as a function of t_μw_ and fit to exponential functions, as explained in Figure 3. The enhancement factors observed are in the ε ≈ 3–14 range, as labeled in each panel.

Table 1 presents a more comprehensive description of these features for all compounds examined in this study, including the observed ^19^F ODNP enhancement factors (ε), the longitudinal relaxation rates of the pure analytes without radical R_1_(pure), and those measured for 40 mM BDPA-doped samples with and without microwave irradiation (R_1_(μw-off) and R_B_(μw-on), respectively). From these relaxation rates, the leakage factor for each system was calculated according to *f* = 1 – *R*_1_(pure)/*R*_1_(μw-off). The obtained leakage factors span the range *f* = 0.43–0.86, consistent with an efficient electron–^19^F cross-relaxation across the various molecules. Assuming a constant saturation factor *s* = 0.63, determined from the 540 mM HFB solution containing 40 mM BDPA with B_1e_ = 0.04 mT, the corresponding coupling factors (ξs) for all measured compounds were also derived (see Table 1). The resulting coupling factors are small, ranging from −0.067 to −0.14.

**Table 1:** Compilation of ^19^F Parameters for Analytes in Liquid-State 14.1T ODNP NMR Studies

Sample	R_1_(pure)	R_1_(μw-off)	R_B_(μw-on)	ε^c^	*f*	ξ
HFB^a^	0.37 ± 0.04	0.91 ± 0.08	0.19 ± 0.03	37 ± 1^d^	0.59 ± 0.08	0.14 ± 0.02
2,4,5-TFBA: 4^a^	0.4 ± 0.1	0.7 ± 0.2	0.11 ± 0.02	28 ± 2	0.4 ± 0.2	0.15 ± 0.08
2,4,5-TFBA: 2,5^a^	0.36 ± 0.06	0.7 ± 0.2	0.13 ± 0.02	31 ± 2	0.5 ± 0.2	0.14 ± 0.06
4,4′-DFBP^a^	0.39 ± 0.02	0.82 ± 0.08	0.15 ± 0.02	29 ± 2	0.52 ± 0.06	0.12 ± 0.02
3′,4′,5′-TFP: 4′^a^	0.41 ± 0.08	1.0 ± 0.1	0.10 ± 0.02	15 ± 1	0.6 ± 0.1	0.05 ± 0.01
3′,4′,5′-TFP: 3′,5′^a^	0.33 ± 0.04	1.1 ± 0.1	0.10 ± 0.01	21 ± 1	0.7 ± 0.1	0.06 ± 0.01
FB^a^	0.09 ± 0.01	0.40 ± 0.05	0.24 ± 0.02	14.8 ± 0.3	0.8 ± 0.1	0.041 ± 0.005
5-FIA^a^	0.28 ± 0.01	0.9 ± 0.1	0.12 ± 0.01	31 ± 1	0.69 ± 0.08	0.10 ± 0.01
2-TFMQ^a^	0.32 ± 0.03	0.9 ± 0.1	0.89 ± 0.07	5.5 ± 0.1	0.64 ± 0.09	0.016 ± 0.002
DEX^a^	0.82 ± 0.09	0.85 ± 0.09	0.75 ± 0.15	4.3 ± 0.4	0.035 ± 0.005	0.21 ± 0.04
HFB^b^	0.25 ± 0.05	1.4 ± 0.2	0.18 ± 0.05	16 ± 1	0.8 ± 0.2	0.04 ± 0.01
4,4′-DFBP^b^	0.22 ± 0.03	1.6 ± 0.2	0.3 ± 0.1	15 ± 1	0.9 ± 0.2	0.036 ± 0.008
5-FIA^b^	0.29 ± 0.04	1.0 ± 0.2	0.3 ± 0.1	19 ± 1	0.7 ± 0.2	0.06 ± 0.02
DEFM^b^	0.19 ± 0.02	1.01 ± 0.17	0.57 ± 0.06	3.4 ± 0.1	0.8 ± 0.2	0.007 ± 0.002
PFD: bridge C–F^b^	0.15 ± 0.01	0.52 ± 0.10	0.43 ± 0.07	12.5 ± 0.6	0.7 ± 0.1	0.037 ± 0.006
PFD: CF_2_ ^b^	0.30 ± 0.02	0.9 ± 0.2	0.52 ± 0.07	4.5 ± 0.7	0.7 ± 0.2	0.012 ± 0.004
PF-1-Hexene: CF_3_ ^b^	0.22 ± 0.02	0.56 ± 0.20	0.40 ± 0.14	12.7 ± 0.9	0.6 ± 0.2	0.04 ± 0.02
PF-1-Hexene: CF_2_ ^b^	0.32 ± 0.04	0.9 ± 0.2	0.38 ± 0.06	14.2 ± 0.7	0.6 ± 0.2	0.05 ± 0.02

a In p-Xylene-d_10_.

b In CCl_4_.

c $\varepsilon =1-\xi fs|\frac{\gamma_{e}}{\gamma_{F}}|=1-700\xi fs$ . ^d^Error derived from spectral sensitivity

Table 1 also presents additional experimental data for HFB, 4,4′-DFBP, and 5-FIA measured in CCl_4_, with details on these alternative solvent measurements presented in the Supporting Information (e.g., Figure S2). Although substantial enhancements are still observed upon replacing *p*-xylene-d_10_ with CCl_4_ (while keeping constant BDPA and solute concentrations and total sample volumes), these εs are approximately halved: HFB goes from 37 ± 1 → 16 ± 1; 4,4′-DFBP goes from 29 ± 2 → 15 ± 1; and 5-FIA from 31 ± 1 → 19 ± 1. We ascribe this primarily due to a decrease in the coupling factor upon going from *p*-xylene-d_10_ to CCl_4_, with | ξ | decreasing from 0.14 → 0.041 for HFB, 0.12 → 0.037 for 4,4′-DFBP, and 0.10 → 0.06 for 5-FIA (assuming identical saturation factors). Simultaneously, the leakage factor increases in CCl_4_ for all analytes (HFB: 0.59 → 0.82; 4,4′-DFBP: 0.52 → 0.86; 5-FIA: 0.69 → 0.71). This suggests that at these fields, an enhanced paramagnetic relaxation but a reduced polarization-transfer efficiency arises in carbon tetrachloride. It seems that the aromatic solvent promotes a more efficient electron–^19^F scalar coupling, likely through BDPA–solute contacts facilitated by π–π interactions between the radicals and the aromatic solvent and/or analyte, or through more suitable time scales to effect a more efficient electron–nuclear cross-relaxation. Importantly, *p*-xylene-d_10_ also offers a superior thermal range thanks to a higher boiling point (138.4 °C) than CCl_4_ (76.7 °C), thereby providing improved tolerance to prolonged microwave irradiation and a more suitable medium for high-power liquid-state DNP experiments.

## 4 Discussion

Overall, this study confirmed the practicality of extending lower-field measurements that based on scalar couplings had shown promise for ^19^F ODNP, all the way to 14.1 T fields. With the aid of gyrotron-based irradiation, a suitable liquid-oriented probehead, and using BDPA as polarizing radical, these experiments could then be successfully performed on relatively large volumes of organic samples, while preserving useful, ≈0.2 ppm resolutions. Enhancements were observed across aromatic and aliphatic ^19^F spectra, with aromatic compounds generally exhibiting larger effects (ε ≈ 15–37) but slower buildups (T_b_ ≈ 4 – 10 s), while aliphatic sites showed more modest enhancements (ε ≈ 3–14) but faster DNP buildups (T_b_ ≈ 1 – 3 s). The coupling factors for the samples tested were generally small (|ξ| ∼ 0.1), leading to modest DNP transfer efficiencies when compared to the theoretically possible maximum (|ξ| = 1). Given, however, that the maximum theoretical electron-to-^19^F ODNP enhancement is on the order of 350, these ξ values explain the still large experimentally observed enhancements.

To obtain a better molecular-level understanding for the pronounced differences in ODNP performance observed between the aromatic and aliphatic ^19^F sites, geometry-optimized structures were calculated for several BDPA-solute systems (Figure 5 and Supporting Information).^100^ For the BDPA/HFB complexes (Figure 5A), calculations consistently converged to configurations in which the HFB ring aligned parallel to one of the aromatic planes of BDPA, forming a π–π stacked arrangement.^90–92^ These stacked orientations suggest an association between the molecules, which would increase an effective electron–nuclear cross-relaxation. A transient interaction driven by π-stacking is consistent with the larger ^19^F ODNP scalar enhancements (20–37×) experimentally observed for aromatic analytes. The situation differs for the BDPA/PF-1-hexene and BDPA/PFD systems (Figure 5B-5C): optimized structures then show no analogous close-contact arrangements between BDPA and the aliphatic fluorines in the hexene or decalin molecules. The larger electron–^19^F separation and the lack of associative interactions such as π–π stacking, would yield a more transient, weakly associated complex.

**Figure 5:**
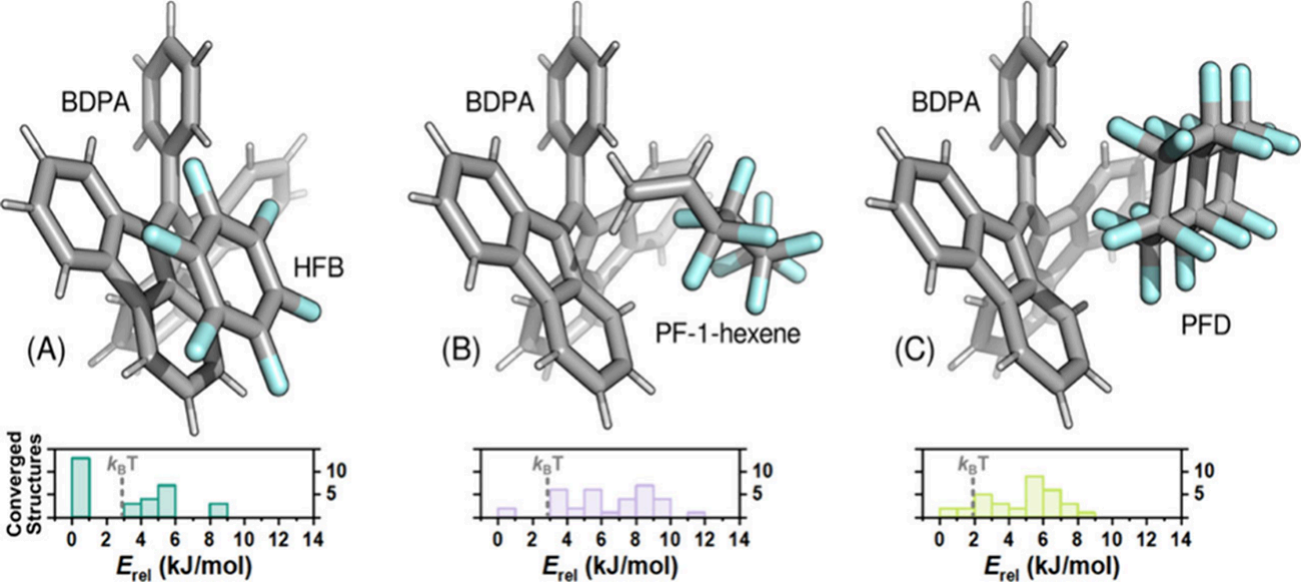
Representative geometry-optimized structures illustrating molecular associations between BDPA and (A) hexafluorobenzene (HFB), (B) 1H,1H,2H-perfluoro-1-hexene (PF-1-hexene), and (C) perfluorodecalin (PFD). For the BDPA/HFB complexes, the optimized geometries show that the aromatic HFB ring preferentially forms π–π stacking interactions with BDPA’s aromatic framework. This configuration should yield relatively short electron–^19^F distances and suitable contact times between the radical and the analyte, consistent with the higher ^19^F DNP enhancement observed for the aromatic systems. (B,C) In contrast, geometry optimization of BDPA/PF-1-hexene and BDPA/PFD complexes reveal fewer close-contact associations between BDPA and the target molecules. Shown underneath each representative structure are the distribution of relative energies resulting from calculations on 33 arrangements for each radical/target pair, illustrating the relatively large fraction of low-energy structures showing association with BDPA for the HFB case.

To further explore this, intermolecular hyperfine couplings to ^19^F were calculated via DFT calculations,^101^ for the aromatic molecule HFB and the aliphatic molecules PFD and PF-1-hexene – each paired with a BDPA radical. Out of the 33 structures calculated for each system, the BDPA/HFB system exhibited an E_rel_ below the thermal energy (E_rel_ < 2.48 kJ/mol) for 13 of the sampled conformers, with a significant cluster of similar-energy conformers at E_rel_ ∼ 0.9 kJ/mol. In contrast, for BDPA/PF-1-Hexene and BDPA/PFD, only 4 and 5 conformers, respectively, showed E_rel_ < 2.48 kJ/mol (Figure 5, lower panels). In line with previous reports for HFB and carbon-based radicals,^48,62^ these calculations suggest that BDPA and HFB tend to form a transient complex stabilized by pi-stacking interactions. As demonstrated in other systems,^90–92^ these transient noncovalent interactions favor higher DNP enhancements, as observed here for the BDPA/HFB pair. Supporting Information Figure S5 expands this insight by describing the hyperfine coupling arising as a function of relative energy, for each of the calculated conformers. Regarding the magnitude of these hyperfine couplings, it is interesting to note that the maximum of these do not correlate with the effective DNP enhancements: (A_iso_)_max_ is 1.1 MHz for BDPA/PF-1-Hexene, 1.8 MHz for BDPA/HFB, and up to 7.7 MHz for BDPA/PFD. This suggests that the dynamics of the radical/analyte pair—which are not accounted for in static simulations—are also critical for defining the efficiency of the spin polarization transfer process.

An additional characteristic observed for the liquid-state ^19^F ODNP of the aromatic sites, was a pronounced increase of the ^19^F *T*_1_ relaxation time under microwave irradiation. We attribute this behavior primarily to the lengthening of *T*_1_ in the extreme-narrowing regime, which arises as the molecular tumbling correlation time *τ*_*c*_ decreases with increasing sample temperature due to microwave absorption. Within the standard Bloembergen–Purcell–Pound (BPP) model,^93^ the nuclear spin–lattice relaxation rate 1/*T*_1_ originates from coupling to fluctuating local magnetic fields generated by molecular motion and is governed by the spectral density function *J*(ω), which depends on *τ*_*c*_:

3 $$\frac{1}{T_{1}}\propto J\left(\omega_{0}\right)=\frac{\tau_{c}}{1+\omega_{0}^{2}\tau_{c}^{2}}$$

In the extreme-narrowing regime where our small molecules and nonviscous systems are present (ω_0_ *τ*_*c*_ ≪ 1), 1/*T*_1_ decreases with faster molecular tumbling, i.e., with decreasing *τ*_*c*_. Consequently, microwave-induced sample heating accelerates molecular motion, leading to an increase of *T*_1_. Arguably this effect is more pronounced in aromatic systems than in aliphatic ones because aromatic analytes can form π–π stacking interactions with the BDPA radical, adducts that exhibit a relatively slow overall tumbling. As microwave irradiation sets in and the sample temperature rises, enhanced Brownian motion disrupts the π–π interactions, leading to a less viscous environment and faster molecular tumbling as well as translational diffusion.^94^ These effects facilitate more frequent encounters between radical and solute molecules, even as their associations become transient. In contrast, aliphatic systems lack such specific π–π stacking interactions with BDPA, and hence the difference in molecular tumbling rates between low- and high-temperature conditions is smaller than in aromatic systems. As a result, the microwave-induced elongation of *T*_1_—and the associated slowing of the DNP polarization buildup rate—is less dramatic for aliphatic analytes. This might explain why microwave-induced heating—although potentially disruptive to BDPA–solute associations and thus detrimental to the Overhauser effect (OE)—does not result in a corresponding decrease in the ODNP plateau with irradiation time (Figures 3 and 4). Rather, two competing processes appear to be at play: on one hand, the disruption of radical–solute associations, and on the other, the enhancement of molecular tumbling and translational diffusion.^94^ These opposing effects may reach a dynamic balance, rendering the ODNP enhancement efficiency only weakly, if at all, dependent on temperature.

For the terminal −CF_3_ groups, the longitudinal relaxation rate without microwave irradiation, R_1_ (0.90 ± 0.10 s^–1^), and the polarization buildup rate under microwave irradiation, R_B_ (0.89 ± 0.07 s^–1^), are essentially identical. This behavior can be attributed to the extremely fast axial rotation of the CF_3_ group about its *C*_*3*_ symmetry axis, resulting in additional relaxation mechanisms such as spin-rotation, that are nearly the same with and without microwave irradiation.

## 5 Conclusions

Although the electron-to-^19^F Overhauser DNP enhancements obtained in this study—up to 37-fold—remain an order of magnitude below the theoretical maximum, these results are still significant in high-field liquid-state DNP. The enhancements were achieved at 14.1 T using relatively large sample volumes in a DNP probe based on a modified conventional liquid-state NMR probe. When using BDPA as polarizing agent, aromatic fluorinated solutes exhibited markedly superior enhancements than aliphatic ^19^F counterparts, reflecting distinct mechanistic regimes governed by electron–nuclear scalar coupling efficiency and different radical–solute interactions. In terms of analytical potential, and considering a near-optimal but realistic scenario whereby the achieved ODNP NMR signal enhancement is ≈30-fold, this would correspond to a potential time saving of about ≈1000 vis-à-vis conventional ^19^F NMR. Still, our experiments required a ca. 10-fold longer recycle delay over conventional counterparts for cooling the sample between microwave irradiation periods, leading to a drop in the SNR/unit_time advantage. In practice, even this gain is further dampened by an excess line broadening induced by the sample heating, leading to ca. 50× gains. Clearly heating is an important outstanding issue to improve, while making sure no other parameter (sample volume, shimming, filling factor) is compromised.

In addition to achieving significant ^19^F signal enhancements, this study confirmed that the established mechanistic understanding of molecular interactions remains valid at high magnetic fields. Specifically, the data demonstrates that the interplay between solvent environment, molecular structure, and radical–solute interactions continue to govern ^19^F ODNP efficiency at 14.1 T, yielding performance that meets or possibly exceeds what has been reported at lower field strengths. The pronounced dependence on structure and solvent confirms that microscopic radical–analyte association mediated by π-stacking, and local environments remain important drivers of spin polarization transfer. These findings validate that existing mechanistic principles can be reliably applied—and potentially leveraged for even greater efficiency—to guide rational solvent selection and molecular design in high-field DNP development.

Although the present high-field, gyrotron-based implementation of liquid-state ^19^F DNP is currently limited to sample systems employing organic solvents with minimal microwave absorption—and is therefore not directly applicable to aqueous sample systems—this approach could still enable substantial gains for detecting and analyzing a diverse range of fluorine-containing compounds. These include pharmaceutical molecules, and the environmental monitoring of persistent and hazardous “forever chemical” species, opening new opportunities for high-field liquid-state DNP in analytical and environmental chemistry.

## Supplementary Material



## Notes

Experimental optimization of the sample; solvent effects; temperature calibrations; nuclear and electron relaxation time measurements; EPR measurements; DFT calculation details (PDF)
