# Supplementary material for: 14.1 T Liquid-State 19F Overhauser Dynamic Nuclear Polarization in an Analytical Organic Setting
Source: J Am Chem Soc. 2026 May 7;148(19):20064–75. doi: 10.1021/jacs.6c03789 (PMC13195678; doi:10.1021/jacs.6c03789)
Supplement: Supplementary file 1 [file ja6c03789_si_001.pdf]

Supporting Information for
14.1 T Liquid-State ^{19}F Overhauser Dynamic Nuclear Polarization in an Analytical Organic Setting

Sungsool Wi,[1*] Jenica Lumata,[1] Thierry Dubroca,[1] Tomas Orlando,[1] and Lucio Frydman[1,2*]

[1] National High Magnetic Field Laboratory, Tallahassee, Florida 32304, USA
[2] Department of Chemical and Biological Physics, Weizmann Institute of Science, 7610001 Rehovot, Israel

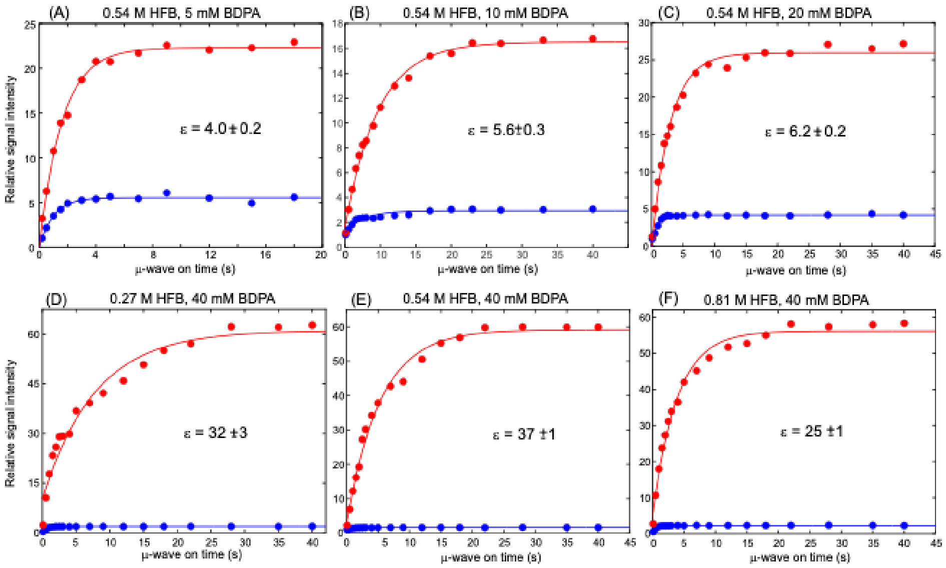

Figure S1. Optimization of BDPA radical and solute (HFB) concentrations for liquid-state ^{19}F Overhauser DNP. Shown are the microwave on/off buildup curves of ^{19}F NMR signal intensities recorded at $\omega_0(^{19}F)$ = 565.083 MHz under the OE condition. (A–C) Fixed HFB concentration of 0.54 M with varying BDPA concentrations of 5 mM, 10 mM, and 20 mM, respectively. (D–F) Fixed BDPA concentration of 40 mM with varying HFB concentrations of 0.27 M, 0.54 M, and 0.81 M, respectively. Red and blue data points represent measurements with microwave irradiation on and off, respectively, and the solid lines are exponential fits to the buildup curves. The resulting DNP enhancement factors (ε) are indicated in each panel. The optimal condition was obtained with 40 mM BDPA and 0.54 M HFB (E), corresponding to approximately 10% v/v solute relative to the solvent.

1. Optimization of the sample and radical concentrations

Optimization experiments for the BDPA radical concentration and the amount of solute molecules are presented in the Supporting Information (Fig. S1). In these experiments, the HFB concentration was fixed at 0.54 M, while the BDPA concentration was varied to 5 mM (Fig. S1A), 10 mM (Fig. S1B), 20 mM (Fig. S1C), and 40 mM (Fig. S1E). Among these, the 40 mM BDPA sample exhibited the largest DNP enhancement. Based on this result, a separate set of measurements was carried out with the BDPA concentration fixed at 40 mM, while varying the solute (HFB) concentration to 0.27 M (Fig. S1D), 0.54 M (Fig. S1E), and 0.81 M (Fig. S1F). As shown in these data, the condition corresponding to Fig. S1E—40 mM BDPA and 0.54 M HFB—yielded the highest DNP enhancement, equivalent to approximately 10% v/v solute relative to the solvent. This optimized composition was therefore employed in all subsequent DNP experiments described in this study. For all measurements,

microwave on/off build-up curves were recorded under the Overhauser-effect (OE) condition at $\omega_0(^{19}F)$ = 565.083 MHz.

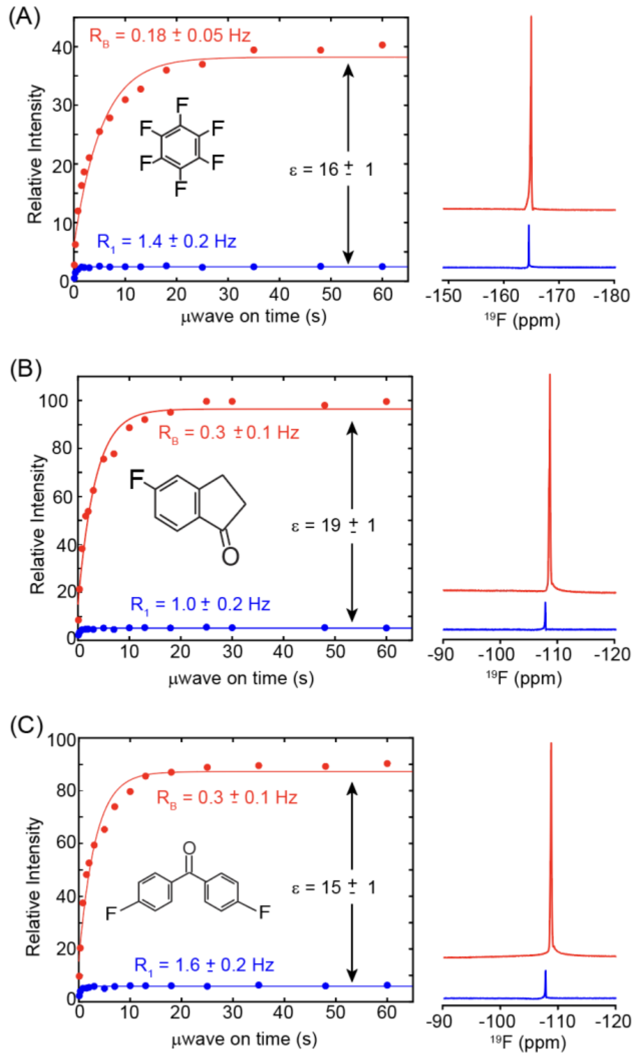

Figure S2. Solvent-dependent liquid-state ^{19}F Overhauser DNP behavior observed for selected aromatic ^{19}F-containing compounds. Shown are the microwave on/off buildup curves (left panels) and corresponding ^{19}F NMR spectra (right panels) recorded at $\omega_0(^{19}F)$ = 565.083 MHz using 40 mM BDPA as the polarizing agent. (A) Hexafluorobenzene (HFB) in CCl_4, (B) 2-fluoro-1-indanone in CCl_4, and (C) 4,4′-difluorobenzophenone in CCl_4. The concentration of each compound in CCl_4 was approximately 10% v/v relative to the solvent. All samples were thoroughly deoxygenated using the freeze–pump–thaw procedure described in the main text. Red and blue data points correspond to measurements with microwave irradiation on and off, respectively, and the solid curves represent exponential fits to the buildup data. The obtained DNP enhancement factors (ε) and relaxation rates (R_B and R_1) are indicated in each panel.

2. Solvent effect on ^{19}F DNP

In Fig. S2, the solvent-dependent behavior of liquid-state ^{19}F Overhauser DNP is examined in detail for three representative aromatic analytes—hexafluorobenzene (HFB), 4,4′-difluorobenzophenone (4,4′-DFBP), and 5-fluoroindanone (5-F-indanone)—which were also presented in Figure 3 of the main text. In this complementary set of experiments, the solvent p-xylene-d_{10} was replaced with carbon tetrachloride (CCl_4) to investigate how solvent effect influences the efficiency of electron–nuclear polarization transfer. For each sample, the BDPA radical concentration was fixed at 40 mM, and both the total sample volume and solute concentrations were kept comparable to those used in p-xylene-d_{10} to ensure a fair comparison. Despite maintaining identical experimental conditions in terms of radical concentration, microwave field strength, and overall sample composition, a clear solvent-dependent effect was observed. Although significant DNP enhancements persisted in CCl_4, their magnitudes were notably reduced compared to those obtained in the aromatic p-xylene-d_{10} medium. Specifically, the enhancement factor (ε) decreased from 37 ± 1 → 16 ± 1 for HFB, from 29 ± 2 → 15 ± 1 for 4,4′-DFBP, and from 31 ± 1 → 19 ± 1 for 5-F-indanone. This systematic reduction—roughly by a

factor of two—highlights the pronounced role of the solvent environment in modulating the DNP mechanism.

As summarized in Table 1, this solvent dependence can be primarily attributed to variations in the coupling factor (ξ), which quantifies the efficiency of polarization transfer between electron and nuclear spins. Assuming that the saturation factor (s) remains identical under both solvent conditions, the coupling factor drops markedly upon changing the solvent from p-xylene-d_{10} to CCl_4: from -0.14 → -0.041 for HFB, from -0.12 → -0.037 for 4,4′-DFBP, and from -0.10 → -0.06 for 5-F-indanone. The substantial reduction in |ξ| in CCl_4 indicates a significantly weaker electron–nuclear scalar coupling interaction, suggesting that the nonpolar, nonaromatic environment of CCl_4 provides less favorable conditions for contact-mediated polarization transfer compared with the π-electron–rich aromatic solvent p-xylene-d_{10}.

3. T_{1e} and T_{2e} measurements of various BDPA radical concentrations

BDPA was dissolved in 30 µL of degassed toluene-d_8 at a concentration of 1-40 mM and loaded into FEP tubes. Electron spin relaxation times (T_1e and T_2e) were measured at 94 GHz (3.35 T) using the high-power, high-field pulsed EPR spectrometer (HiPER) located at the National High Magnetic Field Laboratory (NHMFL, Tallahassee, Florida, USA). T_2e was determined using a variable-delay Hahn echo pulse sequence, while T_1e was measured by incorporating both inversion recovery and saturation recovery methods. Owing to the high peak microwave power available on the HiPER system (>1000 W), 90° pulses of 25 ns duration were employed. The sample temperature was maintained at 27 °C using a helium gas flow. The average microwave power delivered to the sample was About 0.5 W) with a long recycle delay (>100 µs) imposed by the high-power microwave amplifier.

Table S1. T_{1e} and T_{2e} of variable BDPA solutions in Toluene-d_8

BDPA (mM)	T_{2e} (ns)	T_{1e} (µs)[1]	T_{1e} (µs)[2]
1	375	4.0	4.6
2	268	6.5	8.0
5	125	6.4	7.4
10	78.4	2.4	2.0
40	43	-	0.81

[1] T_{1e} measurement with inversion recovery method

[2] T_{1e} measurement with saturation recovery method

4. Temperature Calibration

The sample temperature was evaluated under actual DNP experimental conditions while the sample cell was exposed to microwave irradiation. The microwave-on period was controlled by a shutter synchronized with a 90° pulse following a saturation recovery block, such that irradiation occurred only during the saturation recovery period, while the shutter remained closed during all other intervals, including the signal acquisition delay (d_1). For each measurement with a defined microwave-on duration, the ratio of microwave-on (µ-on) to microwave-off (µ-off) time was

maintained at approximately μ-on/μ-off ≈ μ-on/d_1 = 1/8. A sufficiently long microwave-off period was included in each measurement to prevent excessive sample heating that could lead to solvent boiling. The detailed procedure and corresponding results are summarized in Supporting Figure 3. A nitrogen gas stream flowing at 30 L min^{-1} was supplied to the sample compartment of the probe via an FTS temperature control unit. Initially, an external reference sample of 100% methanol was used to establish the relationship between the FTS set temperature and the actual sample temperature (Fig. S3A). The calibration was based on the known correlation equation:

$$T_{\text{actual}} = -23.832\,\delta^2 - 29.46\,\delta + 403 \qquad \text{(S1)}$$

where $\delta = \delta(\text{CH}_3) - \delta(\text{OH})$(in ppm) represents the chemical shift difference between the CH_3 and OH resonances of methanol. This equation was used to determine the actual temperature corresponding to each FTS temperature setting.

Then, a sample closely resembling the actual DNP conditions was used: a p-xylene-d_{10} solution containing 40 mM BDPA and ^{13}C-labeled 11% CCl_4 + 11% $CHCl_3$, measured without microwave irradiation to establish the correlation between the chemical-shift difference and temperature (Fig. S3B). (Dubroca, Wi, van Tol, Frydman, & Hill, 2019) The nominal FTS temperatures used in (A) were applied, allowing direct conversion to the true sample temperatures determined in (A). A linear correlation between δ = δ(CCl_4) – δ($CHCl_3$) and the actual temperature was obtained as

$$\delta = 0.0023\,T + 18.9026 \qquad \text{(S2)}$$

outside the calibrated range shown in (B). Subsequently, with the FTS temperature set to –30 °C (corresponding to an actual temperature of approximately – 6 °C), microwave irradiation was applied to the sample for durations ranging from 0.1 s to 40 s (Fig. S3C). Microwave-off periods (acquisition delay time d_1) eight times longer than the corresponding microwave-on durations were employed, consistent with the timing scheme used in the actual DNP experiments. The sample temperature increased markedly with longer microwave-on times, rising from approximately – 10 °C to 50 °C for 0.1–5 s irradiation, 60–140 °C for 6–20 s, and exceeding 140 °C for durations beyond 20 s, reaching a maximum of about 160 °C at the longest microwave-on time (45 s) shown in (C). Although the boiling point of p-xylene is approximately 139 °C at atmospheric pressure, the presence of dissolved radical and solute molecules, together with the tightly sealed FEP sample tube (thermally welded at the tip), generated a mildly pressurized environment that effectively suppressed solvent boiling during the experiment. (Neugebauer, et al., 2013).

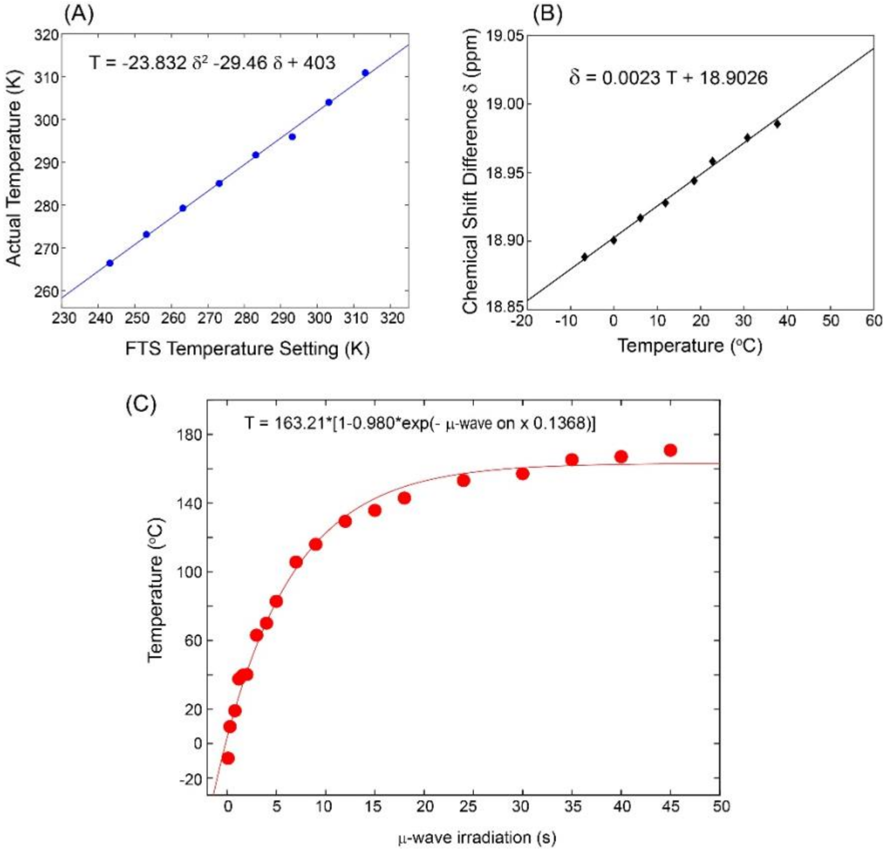

Figure S3. Sample temperature calibration for DNP experiments at an off-OE condition (ν_0 = 564.96 MHz). A nitrogen gas stream flowing at 30 L min^{-1} was delivered into the sample compartment of the probe via an FTS temperature control system. (A) Temperature calibration using an external standard (100% methanol) to establish the correlation between the FTS set temperature and the actual sample temperature. (B) Calibration performed on a sample closely mimicking the actual DNP conditions: a p-xylene-d_{10} solution containing 40 mM BDPA and ^{13}C-labeled 11% CCl_4 + 11% $CHCl_3$, measured without microwave irradiation. The nominal FTS temperatures used in (A) were applied, allowing direct conversion to true sample temperatures determined in (A). (C) Temperature evolution under microwave irradiation (0.04 mT; 13 W) for various microwave-on durations (0.1–40 s), preceded by cooling periods (d_1), with a microwave-on/off duty ratio of 1 : 8. The maximum sample temperature reached after 45 s of continuous microwave irradiation (d_1 = 360 s) was approximately 160 °C.

5. The origin of $m \neq 1$ in Eq. (1)

The longitudinal recovery following incomplete saturation in the saturation-recovery T_1 measurement is described by the general Bloch-equation solution

$$M_z(t) = M_0 + [M_z(0) - M_0]e^{-t/T_1}, \tag{S3}$$

which yields

$$M_z(t) = M_0[1 - me^{-t/T_1}] \tag{S4}$$

when a non-zero residual longitudinal magnetization remains immediately after the saturation pulse block, i.e., $M_z(0) = M_0(1 - m)$, due to incomplete saturation. For $m = 1$, corresponding to complete saturation ($M_z(0) = 0$), Eq. (S4) reduces to the ideal recovery expression

$$M_z(t) = M_0[1 - e^{-t/T_1}]. \tag{S5}$$

6. Variable Temperature T_1 measurement

Variable-temperature T_1 measurements were carried out on a solution containing 540 mM HFB and 40 mM BDPA in *p*-xylene-d_{10} to demonstrate the increase in T_1 with increasing temperature. All experiments were performed in the absence of microwave irradiation. The sample temperature was precisely regulated using the FTS temperature-control system with an N_2 gas flow rate of 30 L min^{-1}, and calibrated using 100% CH_3OH as an external temperature standard. The resulting T_1 (R_1) values are summarized in Table S2.

FTS setting (°C)	Actual temperature (°C)	T_1 (s)	R_1 (Hz)
-30	-1.8	1.10 ± 0.12	0.91 ± 0.08
-5	12.1	1.24 ± 0.17	0.93 ± 0.13
5	16.9	1.34 ± 0.15	0.75 ± 0.10
15	22.9	1.67 ± 0.16	0.60 ± 0.06
30	30.3	1.71 ± 0.12	0.58 ± 0.04
45	38.9	2.20 ± 0.14	0.45 ± 0.03
50	41.7	2.31 ± 0.14	0.43 ± 0.03

7. EPR Methods

The EPR spectra shown in Fig. 2A were fitted with the Matlab package Easyspin and the routine *garlic* (StollS. & SchweigerA., EasySpin: Simulating cw ESR spectra, 2007). The g-tensor [2.00265, 2.00263, 2.00240] was obtained from previous literature. (Dubroca, et al., 2023) The simulations considered a spin S=1/2 coupled with 16 ^{1}H nuclei (I=1/2). The hyperfine coupling tensors utilized in the simulations are reported in Table S2, and were obtained from quantum chemistry calculations with density-functional-theory for the BDPA molecules, as described in the following section. A scale factor up to 20% was introduced in the calculated hyperfine coupling values to fit the experimental spectra. The inhomogeneous broadening due to the hyperfine coupling accounts for most of the observed linewidth, while a Lorentzian linewidth was set at 0.06 mT. The correlation time was fixed at 15 ps, which is a reasonable value for radicals in solutions (Levien, Hiller, Tkach, Bennati, & Orlando, 2020).

Table S2. Hyperfine coupling tensors (A_x, A_y, A_z) obtained from DFT calculations of the BDPA molecule. To obtain a better agreement between the simulation and the EPR spectra, the values were scaled by up to 20%. The resulting A_{iso} used in the simulations is reported in the last column.

label	^{1}H nuclei	A_x	A_y	A_z	scale factor	A_{iso}
A_1	4	-2.67	-7.13	-9.62	1	6.48
A_2	4	-2.28	-6.49	-8.82	1	5.86
A_3	4	1.02	1.64	3.32	0.9	1.99
A_4	2	0.54	1.56	2.72	1	1.61
A_5	2	0.30	1.45	2.28	0.9	1.34
A_6	1	0.73	0.84	1.16	0.8	0.91

8. DFT

To obtain the hyperfine couplings of ^{1}H in the BDPA molecule, we used DFT simulations performed with Orca (Neese, F. Software update: the ORCA program system, version 5.0, WIRES Comput. Molec. Sci., 2022 12(1)e1606. Doi.org/10.1002/wcms.1606). The geometry of the radical was optimized with the functional B3LYP, the basis set def2-TZVPP, and the dispersion correction D3BJ. For the hyperfine coupling, we utilized the basis set EPR-III (V. Barone, in Recent Advances in Density Functional Methods, Part 1, D. P. Chong ed., World Scientific, Singapore, 1995, p. 287). Following a method pioneered by Dorn and coworkers (X. Wang, et al., 2015) and extended by Bennati and coworkers (Reinhard, Levien, Bennati, & Orlando, 2023) (Yang, Orlando, & Bennati, 2025) (Reinhard, et al., 2025), the intermolecular hyperfine coupling to ^{19}F was calculated for three target molecules, namely hexafluorobenzene (HFB), 1-fluorohexene (PF-1-Hexane), and perfluorinated decalin (PFD). Various conformers of the BDPA/target molecule pair were calculated with the software crest. For each BDPA/target molecule system, we selected the 33 conformers with the lowest energy. Each conformer was then optimized with Orca 6.0 utilizing the method R2SCAN-3c. Additional single point calculations were performed on each structure to calculate the single point energy (functional B3LYP, dispersion D4, basis set def2-QZVPP) and the intermolecular hyperfine coupling to ^{19}F (functional B3LYP, dispersion D4, basis set EPR-III). The optimized structures for each system are shown in Figure S4.

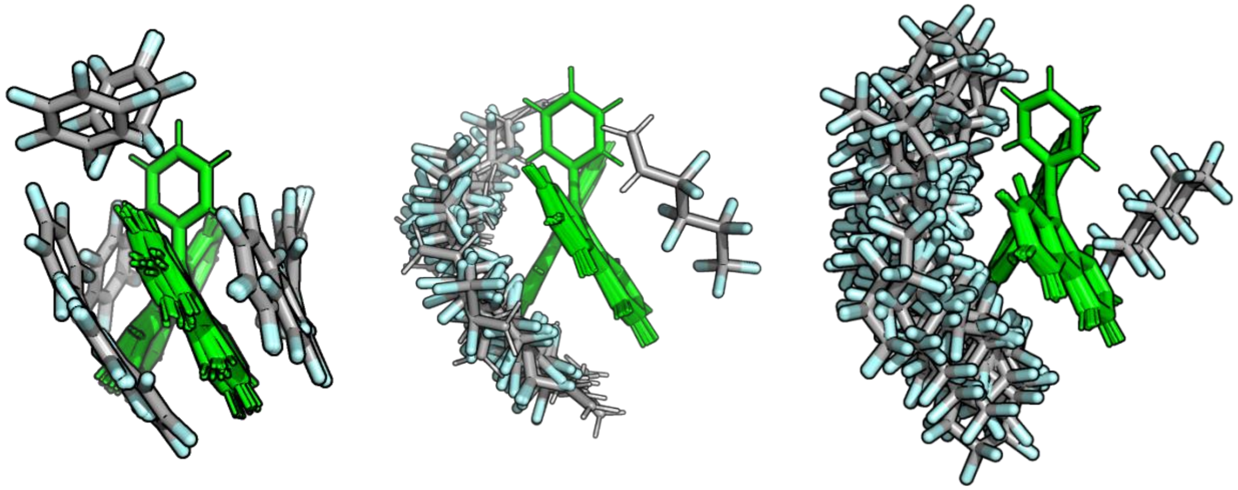

(a) (b) (c)

Figure S4. Overlay of the optimized structures of (a) BDPA/HFB; (b) BDPA/PF-1-hexene; (c) BDPA/PFD.

9. DFT Discussion

The intermolecular hyperfine coupling to ^{19}F was calculated for each conformer and it is shown as a function of the relative energy (E_{rel}) in Figures S5 and S6. Out of the 33 conformers for each system, 13 conformers for the system BDPA/HFB have a E_{rel} below the thermal energy,

E_{rel}<2.48 kJ/mol, with a cluster of conformers with similar energy at E_{rel}~0.9 kJ/mol. On the contrary, for BDPA/PF-1-hexene and BDPA/PFD only 4 and 5 conformers, respectively, show E_{rel}<2.48 kJ/mol. in line with what has been previously reported for HFB and caron-based radicals (Potenza & Poindexter, 1968) (Reinhard, et al., 2025), our calculations suggest that BDPA and HFB have the tendency to form a transient complex that is favored by a pi-stacking type of interaction. As previously shown in various systems (Russ, et al., 2007) (Orlando, Kuprov, & Hiller, Theoretical analysis of scalar relaxation in 13 C-DNP in liquids, 2022) (Reinhard, Levien, Bennati, & Orlando, 2023) (Yang, Orlando, & Bennati, 2025), transient non-covalent interactions favor higher enhancements, as observed here for BDPA/HFB.

Regarding the value of the hyperfine couplings, it is interesting to observe that all systems show maximum values of $|A_{iso}|$ in the order of a few MHz: $(A_{iso})_{max}$ is 1.1 MHz for BDPA/PF-1-hexene, 1.8 MHz for BDPA/HFB, and up to 7.7 MHz for BDPA/PFD (Figures S5 and S6). Contrary to what has been reported for ^{13}C OE-DNP (X. Wang, et al., 2015) (Levien, Hiller, Tkach, Bennati, & Orlando, 2020), here the magnitude of the hyperfine coupling does not correlate with the observed experimental enhancement, suggesting that the dynamics of the radical/analyte pair, which is not accounted for in static simulations, is detrimental for the spin polarization transfer process.

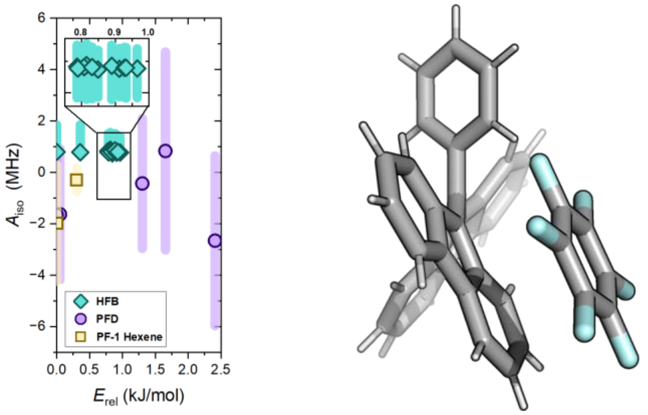

(a) (b)

Figure S5. (a) Intermolecular ^{19}F A_{iso} obtained from DFT calculations as a function of the relative energy for each conformer. The symbol represent the average A_{iso} for each conformer, while the colored band spans from (A_{iso})max to (A_{iso})min. The conformers reported are the ones with relative energy below the thermal energy 2.48 kJ/mol. (b) Optimized structure of the conformer with minimum energy for the system BDPA/HFB.

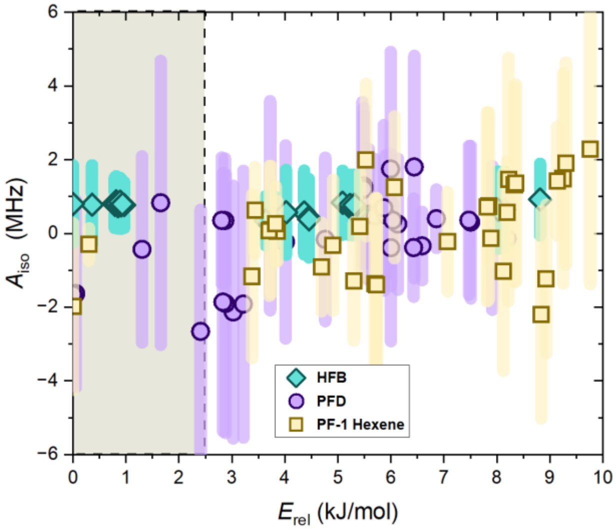

Figure S6. Intermolecular ^{19}F A_{iso} obtained from DFT calculations as a function of the single point energy for each conformer. The symbol represents the average A_{iso} for each conformer, while the colored band spans from $(A_{iso})_{max}$ to $(A_{iso})_{min}$. The gray shaded area represents the conformers with relative energy below the thermal energy 2.48 kJ/mol.

References:

Cruickshank, P. A., Bolton, D. R., Robertson, D. A., Hunter, R. I., Wylde, R. J., & Smith, G. M. (2009). A kilowatt pulsed 94 GHz electron paramagnetic resonance spectrometer with high concentration sensitivity, high instantaneous bandwidth, and low dead time. *Rev. Sci. Instrum., 80*, 103102. doi:https://doi.org/10.1063/1.3239402

Dubroca, T., Wang, X., Mentink-Vigier, F., Trociewitz, B., Starck, M., Parker, D., . . . Krzystek, J. (2023). Terahertz EPR spectroscopy using a 36-tesla high-homogeneity series-connected hybrid magnet. *J. Magn. Reson., 353*, 107.

Dubroca, T., Wi, S., van Tol, J., Frydman, L., & Hill, S. (2019). Large volume liquid state scalar Overhauser dynamic nuclear polarization at high magnetic field. *Phys. Chem. Chem. Phys., 21*, 21200-21204.

Levien, M., Hiller, M., Tkach, I., Bennati, M., & Orlando, T. (2020). Nitroxide Derivatives for Dynamic Nuclear Polarization in Liquids: The Role of Rotational Diffusion. *J. Phys. Chem. Lett., 11*(5), 1629-1635.

Neugebauer, P., Krummenacker, J. G., Denysenkov, V. P., Parigi, G., Luchinat, C., & Prisner, T. F. (2013). Liquid state DNP of water at 9.2 T: an experimental access to saturation. *Phys. Chem. Chem. Phys., 15*(16), 6049-6056.

Orlando, T., Bui, H., Cabigting, J., Ibbetson, N., van Tol, J., Dubroca, T., . . . Mentink-Vigier, F. (2025). Impact of non-polar solvents in dynamic nuclear polarization at high magnetic fields. *J. Magn. Reson., 375*, 107885. doi:https://doi.org/10.1016/j.jmr.2025.107885

Orlando, T., Kuprov, I., & Hiller, M. (2022). Theoretical analysis of scalar relaxation in 13 C-DNP in liquids. *J. Magn. Reson., 10-11*, 100040.

Potenza, J. A., & Poindexter, E. H. (1968). Intermolecular Radical-Solvent Hyperfine Coupling in Fluorocarbons. *J. Am. Chem. Soc., 90*(23), 6309–6317.

Reinhard, M., Levien, M., Bennati, M., & Orlando, T. (2023). Large 31P-NMR enhancements in liquid state dynamic nuclear polarization through radical/target molecule non-covalent interaction. *Phys. Chem. Chem. Phys., 25*, 822-828.

Reinhard, M., van der Ham, A., Yang, L., Bröker, L., Orlando, T., Tkach, I., . . . Bennati, M. (2025). Enhancing NMR Signals in Liquids by Fluorine-19 Overhauser Dynamic Nuclear Polarization (DNP) and Hyperpolarization Transfer to Carbon-13. *Angew. Chem. Int. Ed., 64*(50), e202517498. doi:https://doi.org/10.1002/anie.202517498

Russ, J. L., Gu, J., Tsai, K. H., Glass, T., Duchamp, J. C., & Dorn, H. C. (2007). Nitroxide/substrate weak hydrogen bonding: Attitude and dynamics of collisions in solution. *J. Am. Chem. Soc., 129*, 7018-7027.

Stoll, S., & Schweiger, A. (2006). EasySpin, a comprehensive software package for spectral simulation and analysis in EPR. *J. Magn. Reson., 178*(1), 42-55.

Stoll, S., & Schweiger, A. (2007). EasySpin: Simulating cw ESR spectra. *Biol. Magn. Reson., 27*, 299-321.

X. Wang, W. I., Salido, S. I., Sun, Z., Song, L., Tsai, K. H., Cramer, C. J., & Dorn, H. C. (2015). Optimization and prediction of the electron– nuclear dipolar and scalar interaction in 1 H and 13 C liquid state dynamic nuclear polarization. *Chem. Sci., 6*, 6482-6495.

Yang, L., Orlando, T., & Bennati, M. (2025). Halogen-Bond-Mediated 13C Overhauser Dynamic Nuclear Polarization at 9.4 T. *J. Chem. Phys. Lett., 16*(18), 4505–4514.
